# Novel Nuclear Factor-KappaB Targeting Peptide Suppresses β-Amyloid Induced Inflammatory and Apoptotic Responses in Neuronal Cells

**DOI:** 10.1371/journal.pone.0160314

**Published:** 2016-10-20

**Authors:** Mythily Srinivasan, Baindu Bayon, Nipun Chopra, Debomoy K. Lahiri

**Affiliations:** 1 School of Dentistry, Indiana University–Purdue University Indianapolis, Indianapolis, Indiana, United States of America; 2 Institute of Psychiatry Research, Department of Psychiatry and Medical & Molecular Genetics, School of Medicine, Indiana University–Purdue University Indianapolis, Indianapolis, Indiana, United States of America; Georgia Regents University, UNITED STATES

## Abstract

In the central nervous system (CNS), activation of the transcription factor nuclear factor-kappa B (NF-κβ) is associated with both neuronal survival and increased vulnerability to apoptosis. The mechanisms underlying these dichotomous effects are attributed to the composition of NF-κΒ dimers. In Alzheimer’s disease (AD), β-amyloid (Aβ) and other aggregates upregulate activation of p65:p50 dimers in CNS cells and enhance transactivation of pathological mediators that cause neuroinflammation and neurodegeneration. Hence selective targeting of activated p65 is an attractive therapeutic strategy for AD. Here we report the design, structural and functional characterization of peptide analogs of a p65 interacting protein, the glucocorticoid induced leucine zipper (GILZ). By virtue of binding the transactivation domain of p65 exposed after release from the inhibitory IκΒ proteins in activated cells, the GILZ analogs can act as highly selective inhibitors of activated p65 with minimal potential for off-target effects.

## 1. Introduction

An accumulating body of evidence suggests that a combination of age related changes in the central nervous system (CNS) with excessive or prolonged inflammatory responses contribute to the pathophysiology of neurodegeneration, synaptic dysfunction and hippocampal behavior deficits in conditions such as Alzheimer's disease (AD) [1, 2]. The pleiotropic transcription factor, nuclear factor-kappa B (NF-κΒ) is induced by many physiological and pathological stimuli in the CNS [2–4]. The NF-κΒ family consists of five members, p50, c-rel, p65, RelB and p52 that can diversely combine to form transcriptionally active dimers. It has been suggested that the nature of the dimers determine the effects of activated NF-κΒ. While c-rel containing dimers preferentially promote transactivation of anti-apoptotic factors, activation of p65/p50 dimers primarily enhance inflammatory and pro-apoptotic gene transcription. Positive and negative regulatory mechanisms maintain a balance between the neuroprotective c-rel dimers and the predominantly deleterious p65:p50 dimers in healthy CNS [2, 5, 6].

In AD, secondary stimuli such as accumulating beta amyloid (Aβ) and oxidative stress increase activation of p65:p50 dimers in glial cells [7]. Cleavage of amyloid precursor protein (APP) by beta site amyloid precursor protein cleaving enzyme-1 (BACE-1) is essential for Aβ generation. The promoter region of human BACE-1 gene exhibits κΒ binding elements that physically interact with NF-κΒ p65 [8, 9]. Activation of NF-κΒ p65 increases endogenous BACE-1 transcription and consequent Aβ production [8, 10]. Increased presence of activated p65 and BACE-1 has been observed around Aβ plaques in postmortem AD tissues [11–13]. Extracellular Aβpeptides predominantly activate p65:p50 dimers in glia and post-mitotic neurons and enhance transactivation of inflammatory and pro-apoptotic genes [13–15]. Increased presence of IL-1β, IL-6, and TNF-α have been reported in the affected tissues, serum and CSF of AD patients [16, 17]. Elevated Bax (proapoptotic) to Bcl-2 (anti-apoptotic) ratio have been observed in Aβ stimulated neuronal cells [18, 19]. A feed-back loop of excessive Aβ accumulation, NF-κΒ activation, cytotoxicity and more Aβ production culminate in neurodegeneration [20]. Conditional knock out of p65 has been shown to attenuate BACE-1 transcription and Aβ genesis in AD mice [10]. Absence of p65 co-factors such as p300/CREB binding associated factor has been shown to mediate resistance to Aβ induced toxicity [21]. Thus, although neuronal p65 has been shown to contribute to the physiological functions of synapse formation and transmission, considerable evidence suggest that excessive activated p65 in the CNS lead to neurodegenerative pathology. Hence selective inhibition of activated p65 could suppress AD [2, 16].

Structurally p65 has an amino terminal rel homology domain (RHD), a nuclear localization sequence (NLS) masked by the κΒ inhibitory complex and a carboxy terminal transactivation domain (TAD). The transactivation activity of p65 is mediated by interactions of the TAD with co-regulators and the basal transcription machinery [22, 23]. Glucocorticoid induced leucine zipper (GILZ) is a p65 binding protein that sequesters activated p65 and inhibits transactivation of inflammatory and apoptotic factors [24, 25]. Mutational and binding analyses localized the interaction interface to the proline rich carboxy terminus of GILZ and the TAD of p65 [26]. Molecular modeling suggested that the p65 binding domain of GILZ adopts a flexible polyproline type II (PP_II_) helical conformation that interacts with the highly conserved F^534^/F^542^ in p65-TAD [27].

In recent years, considerable success has been achieved in the development of structurally engineered peptide analogs of the binding epitope(s) of a protein as therapeutic leads [28, 29]. The strategy is increasingly adopted in the design of mimics of proline rich motif that mediate transient intermolecular interactions. The specificity of the interaction is determined by the nature of the proline rich binding domain interface [30, 31]. Here we investigated the efficacy of rationally designed peptide analogs of the p65-TAD binding region of GILZ to selectively sequester activated p65. Structural and functional analyses suggest that select GILZ analog (GA) bind p65-TAD with optimum affinity, exhibit an estimated half minimal lethal dose comparable to known peptide drugs and suppress Aβ_1–42_ induced cytotoxicity.

## 2. Materials and Methods

### Peptides and reagents

All GILZ peptides were synthesized as peptide amides with amino-terminal acetylation (Genescript, Piscataway, NJ) at 95% purity as confirmed by mass spectrometry. To facilitate intracellular delivery the GA were either co-synthesized with the cell penetrating agent, TAT (transactivator of transcription) peptide or used as covalent mixture with Pep-1 chariot peptide (Anaspec, Fremont,CA). Recombinant human p65 protein (r-p65) with DDK tag (catalog number TP320780), purified recombinant human GILZ protein (r-GILZ) with GST tag and biotinylated anti-DDK antibody were from Ori-Gene Technologies Inc., Rockville, MD. Purified Aβ_1–42_ peptide was purchased from American Peptide company (American peptide company, Sunnyvale, CA: Product # 62-0-80 Lot # 1310160T). Aβ_1–42_ peptide stock (1 mg/mL) was prepared in cell culture medium and incubated at 37°C for 24h prior to use in cell cultures [32].

### Comparative modeling

Models of human GILZ and its mimics were built by the CPH models and Geno3D servers using delta sleep inducing peptide (DSIP-PDB:1DIP) as template based on > 90% sequence similarity[33]. While the Geno3D system builds models based on ‘topology mapping, the CPH system uses profile-based alignment as seed for developing energy-minimized homology model [34, 35]. The secondary structure assignment of the GILZ models was independently assessed by the PROSS (Protein dihedral angle-based Secondary Structure assignment) program[36]. Superimposition of the model of each GILZ mimic with experimentally determined PP_II_ helix and wild type GILZ determined the similarity between the structures in terms of root mean square deviation (RMSD). Homology models of p65-TAD was developed similarly using elongation factor eEF3 (PDB:3H7H) as template with which it shares 42% sequence similarity [37].

### GILZ:p65-TAD docking

Models of human GILZ or GILZ mimic and the p65-TAD were applied as probe and target respectively in PatchDock, a geometry based algorithm that yields docked transformations scored on the basis of molecular shape complementarity and atomic desolvation energy [38–40]. Top one thousand solutions were refined using FireDock (Fast Interaction Refinement in molecular docking), a program that optimizes binding of the probe by restricting side-chain flexibility to clashing interface residues. The refined docking solutions were scored based on softened van der Waals interactions, atomic contact energy, electrostatic and additional binding free energy estimations. The top ranked solutions so obtained were further screened using Chimera for interatomic distance of <5Å between the residues of GILZ mimic and the functionally critical residues of p65-TAD [41]. The solution with most contacts was further refined by FlexPepDock using the wild type GILZ:p65-TAD complex with greater than 50% intermolecular residue contacts as reference.

### Binding of GILZ and human r-p65

Previously we observed that the r-GILZ exhibits ten times higher affinity than a 22 residue GILZ peptide for r-p65 [27]. We used similar method to determine direct binding kinetics of GA hexapeptides with human r-p65. High binding ELISA plates were coated with increasing concentrations of r-GILZ (0.5μM to 20μM) or each GA or control peptide (20μM-640μM) and probed with 80μm r-p65 followed by detection with anti-DDK antibody. Absorbance at 650nm was measured with a mixing time of 30s using BIORAD microplate reader. Percent bound p65 was determined considering the binding response of r-GILZ (20μM) with r-p65 as 100%. The dissociation constant of the interaction between the GA or the control peptide and r-p65 was determined by the method of Friguet et al., as described [42, 43]. A fraction of the bound r-p65 (*x*) and the ratio of bound r-p65 to the free GA or control peptide (*y*) was determined by the equations: *x* = (*A*_*o*_ − *A*)/(*A*_*o*_*)*, where *A*_*o*_ is the absorbance of r-p65·anti-p65 complex in the absence of bound GA or control peptide and *y* = (*A*_*o*_−*A*/*A*_*o*_)/(*a*_*o*_− *i*_*o*_)×(*A*_*o*_ − *A*/*A*_*o*_), where *a*_*o*_ is the total concentration of GA or control peptide and *i*_*o*_ is the total concentration of r-p65. *K*_*D*_ for the interaction was determined by the Scatchard equation: *x* = 1 + *K*_*D*_/*y*.

### Cell Titer-Glo (CTG) luminescent cell viability assay

Human neuroblastoma (SK-N-SH) cells cultured in minimal essential medium (MEM) supplemented with 1% fetal bovine serum (FBS) and 1% penicillin (100U/ml)/streptomycin (100μg/ml) were differentiated with 10 μM all-trans retinoic acid for 7 days [32, 44]. After resting for 24h in the low serum medium, the cells were seeded in 24 well (10^5^cells/well) culture plates in fresh medium and incubated with 50μM or 500μM of individual GA or control peptide in non-covalent mixture with Pep-1 or Pep-1 alone for additional 24h. The cultures were then photographed using a phase contrast Leica microscope (Leica Microsystems Inc, Buffalogrove, IL). Subsequently, the cells were harvested, lysed with lysis buffer (M-PER, Pierce) and the lysate was assessed for metabolic activity using the luciferase based CTG assay (CTG, Promega, Madison, WI). Briefly, cell lysate (5μL) in 25μL of phosphate buffered saline was transferred to an opaque white 96-well plate, and then 30μL of CTG assay solution was added. The relative luminescent signal (RLT) was quantified using a Glowmax luminometer (Promega).

### Lactate dehydrogenase (LDH) assay

To detect direct GA-induced cell lysis we performed LDH release assays (Roche Molecular Diagnostics). Human glioblastoma (U373) cells were maintained in MEM supplemented with 10% FBS and 1% penicillin (100U/ml)/streptomycin (100μg/ml) at 37°C in 5% CO_2_-humidified incubators and sub-cultured once or twice a week [32, 45]. Approximately 5 x 10^4^ U373 cells/well were cultured in 96-well plates in the presence of increasing concentrations of individual GA or control peptide from 0.5μM to 500μM. Cells treated with 2% triton-X 100 (Sigma Aldrich, St. Luis, MO) for 10 minutes served as positive control. Untreated cells served as controls for spontaneous LDH-release. Specific LDH-release was calculated according to the following formula: LDH-release % = 100×(GA or control peptide treated cells—untreated cells)/(positive control-untreated cells). The IC_50_ values were extrapolated by logarithmic estimation. The LD_50_ in mg/kg was predicted using the formula, Log (LD_50_) = 0.435x (log IC_50_) + 0.625 [46].

### Detection of apoptosis by flow cytometry

To further assess cell cytotoxicity, the apoptotic effects of individual GA or control peptide was evaluated by the Annexin-V and propidium iodide (PI) dual staining method (Annexin-V-Fluos staining kit, Roche Diagnositics, Mannheim, Germany) [47]. As opposed to apoptotic cells, necrotic cells with ruptured cell membrane take up PI, the DNA binding dye. Thus, cells which take up both fluorochromes are a mixture of apoptotic and necrotic cells, whereas cells that exclude PI but bind Annexin V are (early) apoptotic cells. U373 cells cultured with varying concentrations of individual GA or control peptide (0.5μM to 50μM) for 24h were centrifuged and suspended in 100μl of Annexin V/PI labelling solution (20μl each of Annexin-V-Fluos labelling reagent and PI in 1ml of binding buffer) for 15min at room temperature. After washing the cells were resuspended in PBS:1% paraformaldehyde and analyzed using a FACScan flow cytometer (BD Biosciences, San Jose, CA).

### Functional assays in human primary mixed brain cultures (HFB)

HFB were prepared and cultured as described [48] (S1 and S2 Docs). Briefly, cells were cultured in Neurobasal medium (Invitrogen) without phenol red supplemented with 1xB27, 50mM GlutaMAX, 1xantibiotic cocktail, 5ng/mL recombinant fibroblast growth factor 2 (bFGF) (Invitrogen), and 2μL/mL Normocin (InVivoGen, San Diego, CA, USA). Cells were counted and seeded onto poly-D-lysine (Sigma-Aldrich) coated 24-well plates (Corning, Lowell, MA, USA) at 1.5x10^5^ cells per well and maintained at 37°C in a 5% CO_2_ incubator. Half media changes were performed every 4th day of culture and morphology was monitored via phase contrast microscopy. Culture medium was removed from cells on day 17 (DIV17) and replaced with Neurobasal medium with B27. Appropriate wells were then added vehicle, or individual GA or control peptide (at 40μM or highest LD_50_ concentration) covalently synthesized with TAT or carrier peptide alone for 30min followed by exposure to Aβ_1–42_ at a final concentration of 10μM/ well and incubated for 4h or 48h. Cells and conditioned media were harvested and stored for further analysis. Relative ATP concentration was measured using the CTG kit (Promega) [45]. Data is presented as ΔRLU = RLU of Aβ_1–42_ exposed cells–RLU unexposed cells. Conditioned media collected were assessed for specific cytokines using the OptEIA kits (BD Biosciences).

### NF-κΒ assay

Primary HFB exposed to Aβ_1–42_ and treated with GA or CP as above was harvested at the end of 4h. Nuclear and cytoplasmic fractions were extracted using the CelLytic™ NuCLEAR™ Extraction Kit (Sigma) following manufacturer’s protocol. Five microgram of nuclear extracts was incubated in a 96-well plate coated with oligonucleotides containing the NF-κΒ consensus nucleotide sequence (5’-GGGACTTTCC-3’). The activated NF-κΒ bound to DNA was detected by anti-p65 antibody followed by a peroxidase coupled secondary antibody and substrate using the TransAM kit protocol (Active Motif). Nuclear extracts of Raji cells was used as the positive control [49].

### Quantitative real-time polymerase chain reaction (RT-PCR)

Primary HFB exposed to Aβ_1–42_ and treated with GA or CP as above was harvested at the end of 24h. Total cellular RNA was isolated using Qiagen kit (Invitrogen, Carlsbad, CA) following manufacturer's protocol. Total RNA (2–4 μg) was reverse transcribed using iScript cDNA synthesis kit (Biorad, CA). The concentration of the cDNA was measured at 260 and 280 nm by the Gensys5 model UV-visible spectrophotometer (Thermoelectronic Corp.,CA). Real-time PCR was performed by using the SYBR green/ROX qPCR master mix (SABiosciences, Frederick, MD) according to manufacturer's recommendations on the CFX96 Touch™ Real-Time PCR Detection System (Biorad laboratories, Hercules, California, USA). Each reaction contains 2×12.5 μl of SYBR green Master Mix, 1 μl of 10 μM of primers and 50 ng of the cDNA, to a total volume of 25 μl. The thermal cycling conditions included an initial denaturation step at 50°C for 2 min, 95°C for 3 min, 39 cycles at 95°C for 30 s, annealing temperature at 54°C for 30 s and extension at 72°C for 30s. The primers are F-GAPDH-5′-AAGGTGAAGGTCGGAGTCAAC-3′; R-GAPDH:5′-G GGGTCATTGATGGCAACAATA-3′(102bp); F-IL-1β:5′-AGCTGATGGCCCTAAACAGA-3′; R-IL-1β:5′GGTCGGAGATTCGTAGCTGG-3′ (89 bp); F-IL-6:5′-AATTCGGTACATCCTCG ACGG-3′; R-IL-6:5′-CAGCTCTGGCTTGTTCCTCA-3′ (260bp); F-Caspase3:5′-CATGGAAG CGAATCAATGGACT -3′; R-Caspase 3–5′ TTCCCTGAGGTTTGCTGCAT-3′ (165 bp); F-Caspase 9: 5′-CACTTCCCCTGAAGACGAGTC-3′ and R-Caspase 9: 5′-CTGATGTGCTCACCTGGGAAA-3′ (162 bp). The gene-specific threshold cycle (Ct) for each sample (ΔCt) was corrected by subtracting the Ct for the housekeeping gene glyceraldehyde 3-phosphate dehydrogenase (GAPDH). Untreated controls were chosen as the reference samples, and the ΔCt for all experimental samples was subtracted by the ΔCt for the control samples (ΔΔCt). The difference in each gene-specific threshold between the samples from vehicle treated and GA or CP treated cells was determined to obtain the relative change in the specific mRNA. The magnitude of change in the mRNA was expressed as 2^ΔΔCt^. Each measurement of a sample was conducted in duplicate.

### Statistical analysis

Statistical significance was assessed by one-way analysis of variance (ANOVA) followed by Tuckey post hoc. A value of p < 0.05 was considered significant.

## 3. Results

### Design and modeling of GILZ mimic

**Fig 1:**
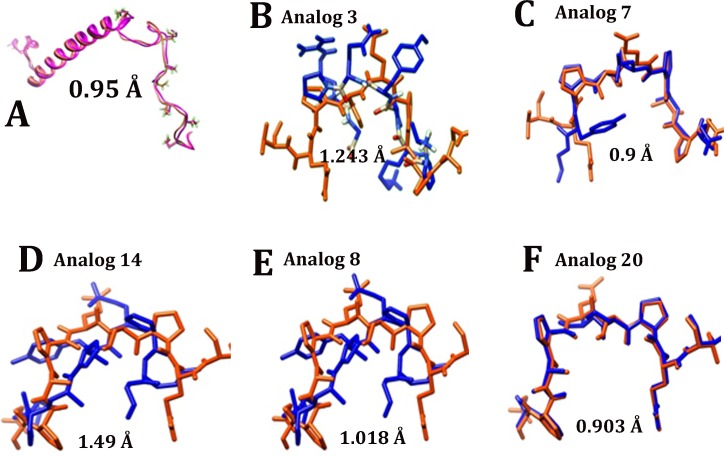
Comparative modeling of GILZ analogs: Superimposition of human delta sleep inducing peptide (DSIP; PDB: 1DIP) with the predicted model of human GILZ (A), superimposition of indicated analog (blue) with the critical residues in the proline glutamic acid rich region of human GILZ model (red) (B-F). Structural similarity in terms of root mean square deviation (RMSD) for each superimposition is indicated.

Strategies to determine the smallest biologically active fragment of a lead peptide involves truncation, deletion, alanine scanning and substitution of residues[29]. Deletion studies suggested that the amino terminal helix of GILZ is critical for dimerization but is not involved in the interaction with NF-κΒ. Truncation mutants suggested that the residues spanning ^120^P-P^127^ of the GILZ were critical for GILZ mediated inhibition of NF-κΒ transactivation [24, 26]. Molecular modeling showed that the GILZ-COOH or a 22 residue peptide derived from the proline rich region adopted an extended PP_II_ helical conformation and interacted with the p65-TAD exhibiting weak binding kinetics [27]. Alanine scanning mutagenesis suggested that the substitution of the ^120^PXXP^123^ motif abrogated the ability to inhibit NF-κΒ transactivation potentially due to loss of PP_II_ conformation[24, 25]. In human and mouse GILZ, hydrogen bonding between the side chain of Ser or Thr and the backbone carbonyl of Glu or Pro respectively could contribute to the stability of PP_II_ conformation [50]. We designed 40 GILZ mimics by incorporating rational substitutions in the p65 binding motif of GILZ with residues that increase the propensity for PP_II_ helical conformation and stabilize it. Comparative modeling with substituted residues for all 40 GA was performed to obtain structural representation of each with reference to the adjacent residues of human GILZ. In addition we introduced conformational constraints by superimposing each GILZ mimic model on structures with solved or experimental PP_II_ helix and wild type human GILZ to select for mimics with significant structural homology (Fig 1). The PP_II_ content of GILZ mimics as determined by PROSS ranged from 14.3%, 28.6% and 42.9%. Since PP_II_ helix formation is a locally driven event with little/no involvement of long-range interactions [51, 52], it is logical to presume that the synthetic GILZ mimetic peptide with blocked end groups will adopt a similar conformation as in the predicted model. Twenty GILZ mimics that exhibit near structural congruence with DSIP (<1Å) or wild type GILZ or experimentally determined PP_II_ structure (<2Å) were selected for *in silico* docking.

### Docking of GILZ mimic and p65-TAD

To be of potential therapeutic value, the GILZ mimic should adopt PP_II_ helical conformation in the context of the critical binding residues in p65-TAD. The p65-TAD is commonly divided into two distinct regions, TAD-1_521−551_ consisting of 36 amino acids and TAD-2_428−520_ with 92 residues [23]. It has been reported that the TAD_1_ accounts for nearly 95% of the transactivation potential of full-length p65 and that the TAD_2_ alone is less potent mediating about 30% activation [22, 53]. In particular, the highly conserved aromatic residues (F^534^, F^542^), acidic residues (D^531^,D^533^) and phosphorylation sites (Ser^529^,Ser^536)^ in p65-TAD_1_ have been identified as critical for transactivation [54].

**Fig 2:**
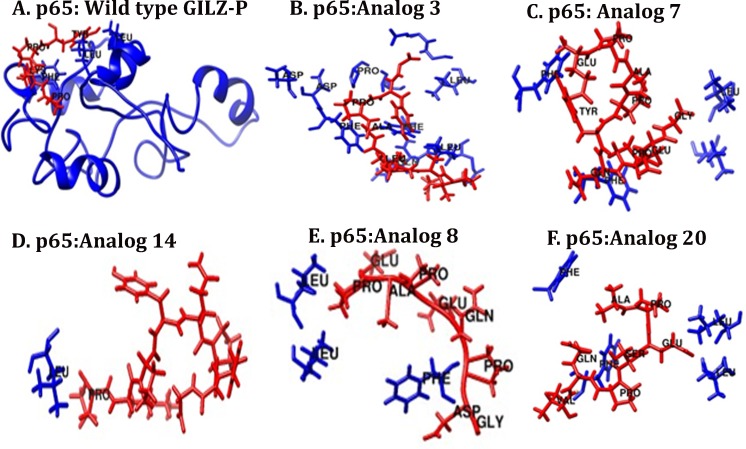
The docked complex of wild type GILZ-PER (proline glutamic acid rich region) or GILZ analog (GA) with human p65-TAD. Representative molecular model of p65-TAD docked (blue) with wild type GILZ-PER (A) and indicated analog (B-F) (red) are shown. The residues in each analog <5Å distance of highly conserved residues in p65-TAD_2_ (Phe or Phe) and the “LXXLL” motif in p65-TAD_1_ that suggest proximity with residues critical for transcriptional activity are shown.

Homology model of p65-TAD was built using solution structure of the elongation factor eEF3 (PDB: 2XI3, 2WI3) with which it shares 42% sequence similarity[37]. The spatial orientations of wild type GILZ and top 20 GILZ mimics with p65-TAD were assessed by multiple docking algorithms. One thousand interaction possibilities identified by rigid-docking algorithm were improved by coarse refinement to restrict side-chain flexibility at the interface. The docked complexes were ranked using an optimized global energy function for higher probability prediction. Top ten solutions of each GILZ mimic were evaluated for proximity to p65-TAD residues. In general two residues are considered in contact with each other if the distance between the Cβ atoms is <5Å [55]. Interactions between the conserved F^534^/F^542^ in the p65-TAD_1_ and the critical prolines P^120^/P^123^ of GILZ could promote C–H··π interaction and provide substantial binding energy in the GILZ:p65-TAD complex. All solutions that exhibited an RMSD of <5Å with the critical F^534^ and F^542^ were selected for further screening (Table 1). Sixty of the top one hundred predictions of wild type GILZ exhibited close proximity with nearly 50% of p65-TAD_1_ residues suggesting near-native interactions (Fig 2A). The wild type GILZ:p65-TAD complex with the lowest global energy and maximum contacts with p65-TAD was selected as reference for refining each of the 20 GILZ mimic-p65TAD complexes in two hundred independent FlexPepDock simulations. Significantly ten of the twenty GILZ mimics exhibited interatomic distance of <5Å not only with the conserved phenylalanine in p65-TAD_1_ but also with the putative LXXLL motif in p65-TAD_2_ (Fig 2B and 2C). The LXXLL motif commonly observed in transcription factors are known to mediate protein–protein interactions [56]. A rank order was developed based on percent PP_II_ content, structural similarity with wild type GILZ and percent contact with p65-TAD. We selected two GILZ mimics or analogs (GA-1 and GA-2) that exhibit near native docking, structural congruence with experimental PP_II_ and wild type GILZ and good PP_II_ potential for screening by cellular analyses. In addition we selected one mimic with good PP_II_ potential but fewer contacts with p65-TAD and two GILZ mimics that possess low PP_II_ content but acceptable percent contact with the p65-TAD in docking analyses as control peptides for PP_II_ conformation (control peptide 1) and p65 docking potential (control peptide 2 and control peptide 3) respectively.

**Table 1:** Characteristics of twenty GILZ mimics.

		Superimposition		p65-Transactivation Domain			
	% PPII	PPII	GILZ model	% TA1	%TA-2		
					CR-1	CR-2	CR-3
				521–531	435–455	462–479	491–505
GA-1	14.3	0.82	0.216	19	43	39	20
GA-2	42.9	0.531	0.216	18	32	13	9
GA-3	14.3	0.109	0.428	11	14	23	4
GA-4	14.3	1.049	0.482	4	13	47	29
GA-5	28.6	1.234	0.586	11	14	23	4
GA-6	14.3	0.844	0.645	21	54	34	9
GA-7	14.3	0.156	0.681	30	20	0	11
GA-8	14.3	1.72	0.842	15	41	32	5
GA-9	42.9	0.257	0.88	24	42	29	13
GA-10	28.6	0.161	0.9	12	38	23	7
GA-11	28.6	0.703	0.912	20	46	23	6
GA-12	42.9	1.17	1	10	12	18	6
GA-13	14.3	0.58	1.018	3	35	55	6
GA-14	28.6	1.172	1.033	32	28	10	2
GA-15	14.3	0.391	1.047	12	36	8	7
GA-16	14.3	0.252	1.154	18	38	33	1
GA-17	28.6	1.293	1.227	15	25	24	9
GA-18	14.3	1,248	1.243	16	24	31	22
GA-19	28.6	0.581	1.49	7	22	24	7
GA-20	42.9	1.174	1.613	37	43	9	2

GILZ models (GM) with substituted residues at the proline rich region of wild type GILZ sequence were developed using Geno3D and CPH Models. Each GA model was superimposed over wild type GILZ and experimentally determined polyproline (PP_II_) helical structures. The root mean square deviation (RMSD) of each superimposition as a measure of structural similarity is shown. Each GM was docked with the molecular model of p65-TAD and the docked complexes were screened for interface p65-TAD residues within 5Å distance of GM.

### Kinetics of GA:-p65 interaction

**Fig 3:**
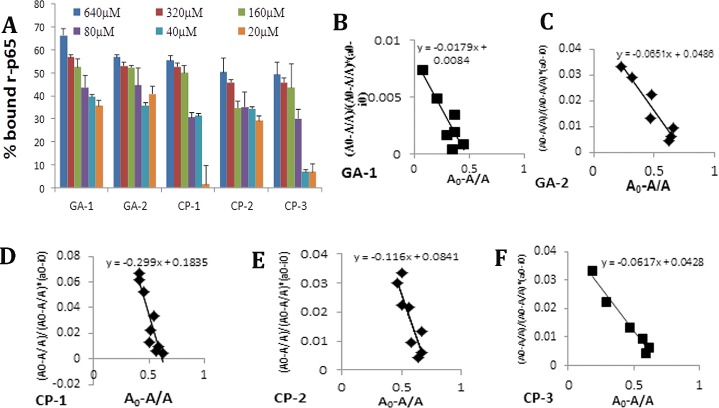
GA-rp65 binding analysis: Binding between the plate-bound GILZ analog (GA) or control peptide (CP) (20μM-640μM) at increasing concentration and the r-p65 was detected with the anti-DDK as described in the methods section. A dose dependent decrease in percent bound r-p65 was observed in association with the GA (A). Data represent average+/-SD from three experiments. Scatchard plot analysis of bound p65 (A_0_-A/A_0_) against the ratio of bound p65 to free GA (y = (A_o_-A/A_o_)/[(A_o_-A/A_o_)* (a_o_-i_o_)] was used to determine the dissociation constant for the interaction between indicated GA and r-p65 (B-F).

Previously the strength of interaction between rGILZ or wild type GILZ-P and r-p65 has been shown to be 5.91±2.4×10^−7^M and 1.12 ± 0.25×10^−6^M respectively[27]. We evaluated the binding kinetics of individual GA at increasing concentrations with the plate bound r-p65 protein at constant concentration. We observed that the percent bound r-p65 was over 25% with GA-1, GA-2 and CP-1 even at the lowest concentration evaluated (Fig 3A). The dissociate constant, K_D_, as calculated by the method of Friguet et al. was 2.29+/-0.2x10^-6^M for GA-1 and 3.24+/-0.19x10^-6^M for GA-2. The strength of interaction of the GA:p65-TAD binding is consistent with other transient protein:peptide interactions such as that of peptide inhibitors of matrix metalloproteinase and that of SMRT peptides binding the BTB domain of BCL6 [57, 58]. The control peptides exhibited higher K_D_ values of 3.27+/-1.8x10^-6^M, 3.4+/-0.15x10^-6^M and 4.28+/-0.5 x10^-6^M for CP-1, CP-2 and CP-3 respectively (Fig 3B–3F).

### Effect of GA on cellular morphology and metabolic activity

**Fig 4:**
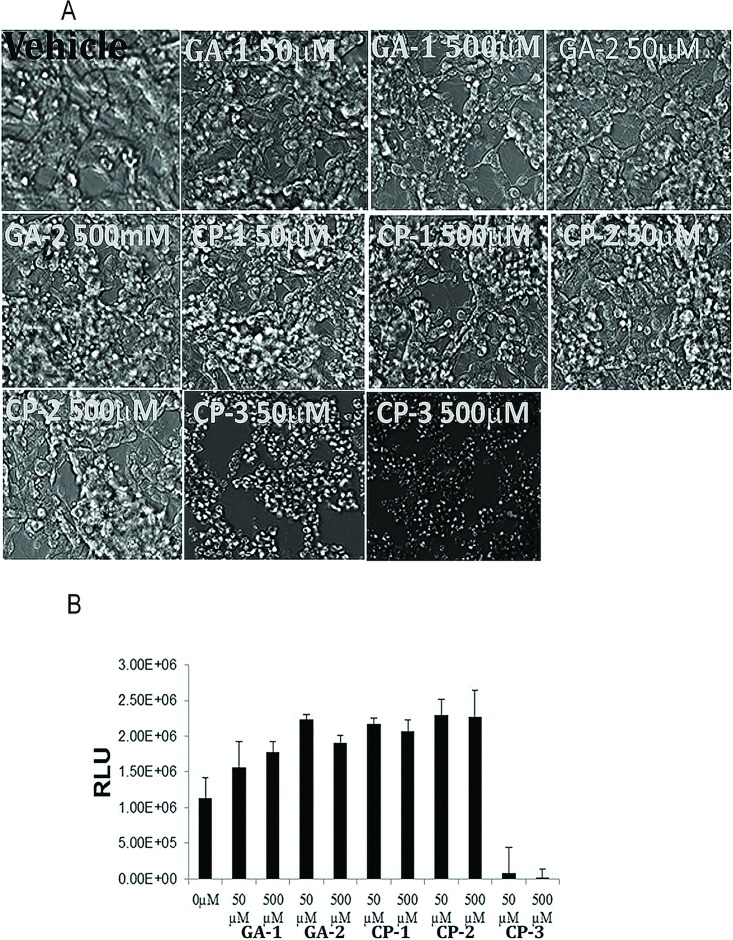
Effect of GILZ analog (GA) on cellular morphology and metabolic activity in neuroblasts: Differentiated SK-N-SH neuroblastoma cultures were exposed to individual GA or control peptide (CP) at the indicated concentration for 24h. (A) Phase contrast imaging of the cells shows no apparent adverse effects on morphology of cells exposed to GA-1 or GA-2 or CP-1 or CP-2 at either concentration. Exposure to CP-3 at both concentrations showed morphological changes consistent with cell death. (B) Cell lysates were assessed by CTG assay to determine relative levels of intracellular ATP. An increase in Relative Luminescence Units (RLU) suggesting cellular viability was observed with all treatments except CP-3. Data represent average+/-SD from three experiments.

Any compound with potential therapeutic effect should be biocompatible and nontoxic. So, we first screened the effects of GA on cellular morphology and viability. Cultures of neuroblasts exposed to 50μM or 500μM of individual GA or CP-1 or CP-2 did not show any change in cell morphology suggesting that the four peptides were not toxic. Exposure to CP-3 at either concentration showed morphological changes consistent with cell death (Fig 4A). The effect of GA on cellular metabolic activity was assessed by the CTG assay which measures intracellular ATP concentrations as an indicator of actual cell number. The results of the CTG assay were very similar and showed that the two GA and CP-1 and CP-2 did not adversely affect the differentiated neuroblastoma cells, but treatment with CP-3 reduced the viability of the cells at both concentrations tested (Fig 4B).

### Effect of GA on membrane integrity and apoptosis

**Fig 5:**
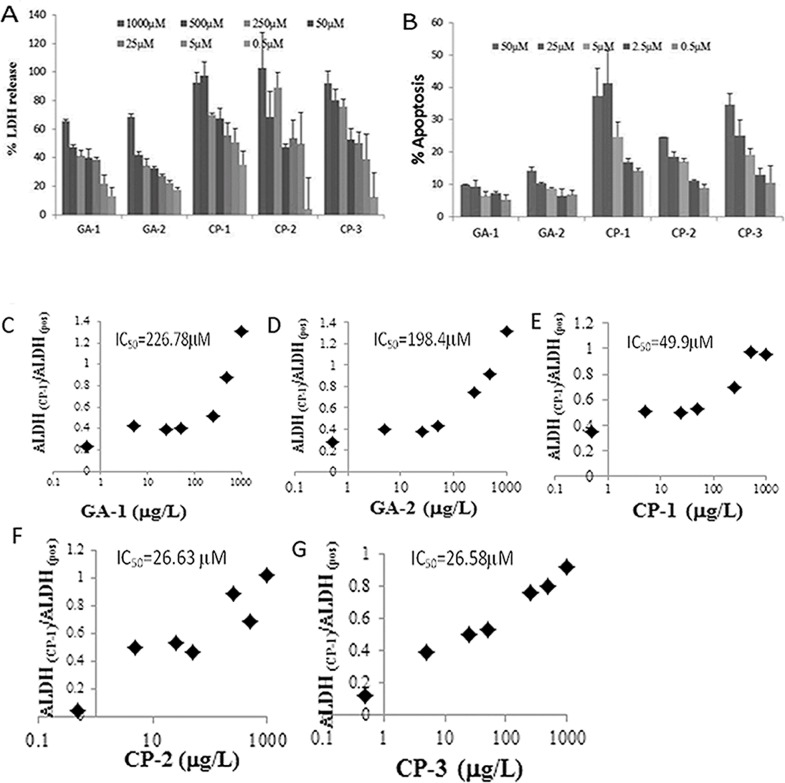
Effect of GA on lactate dehydrogenase (LDH) release: U373 MG astroglioma cells were exposed to increasing concentrations of indicated GILZ analog (GA) or control peptide (CP) (0.5μM to 1mM) for 24 h. The release of LDH into the cell culture supernatant from damaged cells was measured. % LDH was calculated as the ratio of the difference between the peptide treated and untreated cells to that of the difference between the positive control and the untreated cells (A). Data represent average +/- SD from three experiments. The IC_50_ was determined by logarithmic extrapolation (C-G). Flow cytometric analysis of Annexin positive U373 cells treated as indicated was determined as a measure of apoptosis (B).

Membrane integrity as a measure of cell survival was assessed by the leakage of intracellular LDH molecules into culture medium [44]. Treatment with GA-1 and GA-2 were best tolerated as indicated by the reduced % LDH release at all concentrations as compared to untreated cultures or cultures exposed to control peptides (Fig 5A). IC_50_ extrapolated from regression analysis is 226.78 μM for GA-1 and 198.4 μM for GA-2 (Fig 5C and 5D). The IC_50_ was lower for CP-1 (49.9 μM), CP-2 (26.58 μM) and CP-3 (26.63 μM) (Fig 5E–5G). Using the Speilmann method, the LD_50_ in mg/kg body weight is estimated to be 19.78 for GA-1 and 18.66 for GA-2 and 10.2, 7.78 and 7.79 for CP-1, CP-2 and CP-3 respectively [46]. Many therapeutic peptides such as leuprolide and glatiramer acetate have been shown to exhibit similar LD_50_ values[59, 60]. Furthermore the cytotoxic effect of each peptide at increasing concentration from 0.5μM to 50μM was assessed by Annexin and PI staining (Fig 5B). GA-1 and GA-2 exhibit < 25% apoptosis at highest concentration.

### Select GA protects against Aβ_1–42_ induced toxicity in human fetal brain cells (HFB)

**Fig 6:**
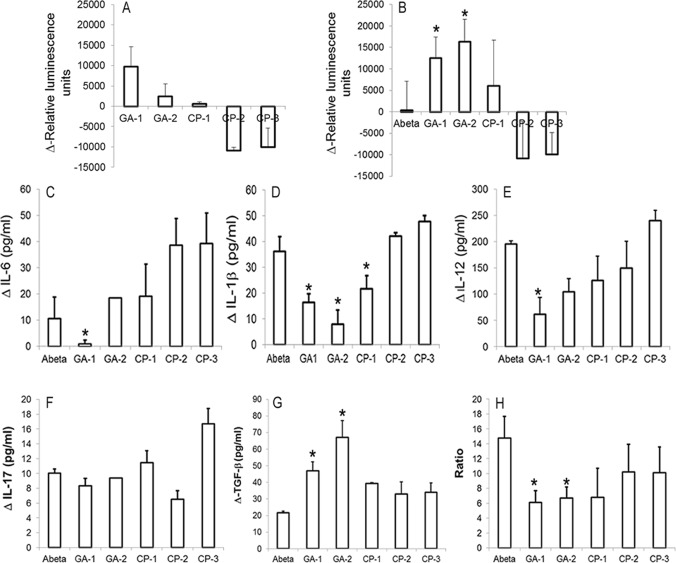
Effect of GILZ analog (GA) treatment on human fetal brain cells (HFB): (A,B): Select GA decrease metabolic activity in Aβ_1–42_ exposed HFB. Primary cultures of HFB (Div 17) were exposed to Aβ_1–42_ and treated with 50M of indicated GA or control peptide (CP). Cytoplasmic extracts of cells collected at 24h was assessed for viability by CTG assay. Data are presented as ΔRLU (difference in relative luminescent units (RLU) between the Aβ_1–42_ exposed cells and unexposed cells) (A, B). GA suppresses inflammatory cytokines in activated HFB. HFB cells were cultured as above and culture medium collected at 24hrs was assessed for pro-inflammatory (IL-1β, IL-6 and IL-17) (C, D, E and F) and anti-inflammatory (TGF-β) (G) cytokines. (H) Effect of GA on NF-κΒ activation. Primary cultures of HFB exposed to Aβ_1–42_ (10μg/ml) and treated with indicated GA or CP as above were harvested at the end of 4 h. 5μg of nuclear extract was tested for binding of the activated p65 NF-κΒ subunit to an NF-κΒ consensus sequence using the Trans AM NF-κΒ ELISA kit. The p65 DNA binding activity was calculated as the ratio of absorbance from Aβ_1–42_ stimulated cells to that of unstimulated cells. Values are the average/-S.D from two experiments. * = p<0.05 as compared to Aβ_1–42_ exposed cells, @ = p<0.05 as compared with Aβ_1–42_ and CP-1 or CP-2 treated cultures.

**Fig 7:**
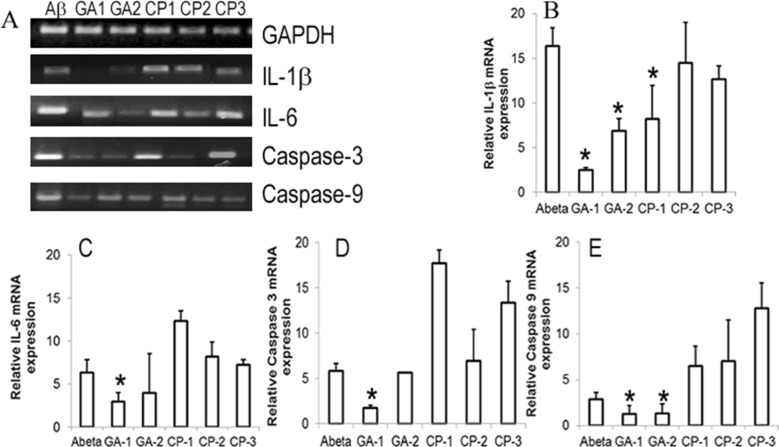
Effect of GILZ analog (GA) treatment on apoptosis related molecules in human fetal brain cells: Primary cultures of HFB were exposed to Aβ_1–42_ and treated with 50μM of indicated GA or control peptide (CP). Total RNA was isolated from cells harvested after 24h and assessed for mRNA for IL-1β, IL-6, Caspase-3 and Caspase-9 by quantitative PCR. The mRNA expression in each sample was finally determined after correction with GAPDH expression. (A) Gel electrophoresis of the PCR products GAPDH (111bp), IL-1β (89bp), IL-6 (260bp), Caspase-3 (165bp) and Caspase-9 (162bp). (B) Relative mRNA quantitation of IL-1β (B), IL-6 (C), Caspase-3 (D) and Caspase-9 (E) with respect to that of housekeeping gene GAPDH is shown. Data are average of two experiments and expressed as means ±SD. * = p<0.05 with respect to vehicle treated cells.

We used an *in-vitro* neurodegeneration model in which primary human fetal brain cells are allowed to mature gradually. The system provides an opportunity to test the pharmacological and toxicological effects of GA on differentiated neurons and glia simultaneously[48]. The relative ATP concentration of GA or control peptide treated HFB cultures in the presence or absence of Aβ_1–42_ over the cultures exposed to vehicle alone (Δ-RLU) was determined by the CTG assay. Cultures exposed to GA were best tolerated while the cultures treated with CP-2 or CP-3 exhibited significant toxicity (Fig 6A). The mean RLU of vehicle treated cells varied between 7352.75+/-1265.2 and 16157.5+/-4950 and the average RLU of cultures exposed to Aβ_1–42_ varied between 6067.25+/-903.05 and 11574.25+/- 4139.3 in different experiments. The viability was significantly higher in cells exposed to Aβ_1–42_ and treated with GA-1 or GA-2 (Fig 6B). Although Δ-RLU was higher in cultures treated with CP-1, it was not significant when compared to that in untreated Aβ_1–42_ exposed cultures. The relative concentration of IL-1β was lower in culture medium of cells exposed to Aβ_1–42_ and GA-1 (16.4 +/-3.3pg/ml) or GA-2 (30.5+/-7.9 pg/ml) treated cells as compared with control peptide treated (CP-1:21.7+/-5.03pg/ml, CP-2: 42.1+/-1.3pg/ml, CP-3: 47.8+/-4.4pg/ml) or untreated (36.1+/-5.6pg/ml) cells. Similarly IL-6 and IL-12 secretion was lower in GA-1 (0.9+/-1.4pg/ml and 61.6+/-32.2pg/ml respectively) or GA-2 (18.4+/-0.1pg/ml and 104.5+/-24.8pg/ml respectively) treated cells as compared with CP-1 (19.17+/-12.2pg/ml and 125.7+/-46.3pg/ml respectively), CP-2 (38.6+/-10.2 pg/ml and 149.9+/-50.7pg/ml respectively), CP-3 (39.2+/-11.7pg/ml and 239.9+/-19.4pg/ml respectively) treated or untreated (10.7+/-1.5pg/ml and 104.5+/-24.8pg/ml respectively) cells (Fig 6C–6E). The relative concentration of IL-17 did not differ between cells treated with GA-1 (8.3+/- 1pg/ml) or GA-2 (9.4+/-0.01pg/ml) peptide and control peptide (CP-1:11.4+/-1.6pg/ml, CP-2: 6.5+/-1.2pg/ml and CP-3: 16.7+/-2 pg/ml) or untreated cells (10 +/-0.6 pg/ml) (Fig 6F). The relative concentration of TGF-β was significantly upregulated in cells treated with GA-1 (46.9+/-5.4pg/ml) or GA-2 (67.1+/-10.2pg/ml) as compared with untreated cells (21.7+/-0.7pg/ml). The relative concentration of TGF-β measured 39.2+/-0.7pg/ml, 32.9+/-7.3pg/ml and 33.9+/-5.6pg/ml in CP-1, CP-2 and CP-3 treated cells respectively (Fig 6G). Further exposure to Aβ_1–42_ resulted in upregulation of mRNA for IL-1β and IL-6. The relative expression (fold increase) of IL-1β and IL-6 mRNA was reduced in cells treated with GA-1 (2.5+/-0.23 and 2.9+/-1 respectively) or GA-2 (6.9+/-1.4 and 4+/-4.5 respectively) as compared with CP-1 (8.2+/-1.7 and 12.9+/-1.2 respectively), CP-2 (14.4+/-4.6 and 8.2+/-1.7 respectively) CP-3 (12.6+/-1.5 and 7.2+/-0.6 respectively) or untreated (16.4+/-2 and 6.3+/-1.5 respectively) cells (Fig 7A–7C).

### Select GA treatment inhibits activated p65

Previously Aβ_1–42_ has been shown to enhance expression of activated NF-κΒ in glia and post-mitotic neurons [14, 15]. We measured nuclear p65 using activated NF-κΒ specific ELISA and calculated the p65 DNA binding activity as the ratio of absorbance form Aβ_1–42_ stimulated cells to that of unstimulated cells. Nuclear p65 was significantly higher in cells exposed to Aβ_1–42_ (14.75+/-3.5) than in unexposed cells (2.5+/-1.7). There was a trend towards decreased nuclear p65 in Aβ_1–42_ exposed cells treated with GA-1 (6.13+/-1.3) and GA-2 (6.7+/-1.5) as compared to untreated cells (14.75+/-3.5). Nuclear p65 of Aβ_1–42_ exposed cells treated with CP-1, CP-2 and CP-3 measured 6.8+/-3.9, 10.2+/-2.9 and 10.1+/-3.7 respectively (Fig 6H). No significant difference in nuclear p65 was observed in cells treated only with the peptides in the absence of exposure to Aβ_1–42_ (data not shown). Similar assessment showed no significant difference in the cytoplasmic NF-κΒ p65 between GA treated and untreated or CP treated cells (data not shown). Although we observed slightly elevated nuclear p50 in Aβ_1–42_ (6.15+/-1.2) than in unexposed cells (0.5+/-2.7), no difference was observed between GA peptide of CP treated cells and untreated cells (data not shown).

### Select GA protects against Aβ_1–42_ induced apoptosis related markers in human fetal brain cells (HFB)

We next determined the message for apoptosis relevant molecules Caspase 3 and Caspase 9 by quantitative PCR. As shown in Fig 7, exposure to Aβ_1–42_ resulted in upregulation of mRNA for caspase 3 and caspase 9 in HFB. The relative expression (fold increase) of IL-1β and caspase 9 mRNA was reduced in cells treated with GA-1 (2.5+/-0.23 and 1.3+/-0.9 respectively) or GA-2 (6.9+/-1.4 and 1.3+/-1 respectively) as compared with CP-1 (8.2+/-1.7 and 6.5 +/-2.2 respectively), CP-2 (14.4+/-4.6 and 7.1+/-4.5 respectively) CP-3 (12.6+/-1.5 and 12.8+/-2.8 respectively) or untreated (16.4+/-2 and 2.9+/-0.7 respectively) cells (Fig 7A, 7B and 7E). Further the IL-6 and Caspase 3 mRNA exhibited significant reduction in GA-1 (2.9+/-1 and 1.7+/-0.3 respectively) treated cells as compared to that in CP-1 (12.9+/-1.2 and 17.7+/-1.5 respectively) treated or untreated (6.3+/-1.5 and 5.8+/-0.9 respectively) cells (Fig 7A, 7C and 7D).

Comparative analysis of physical and functional characteristics of known receptor antagonists and peptide drugs in clinical use today with that of GA, suggest that the GA-1 and GA-2 exhibit significant drug like properties (Table 2).

**Table 2:** Physical and functional characteristics of GILZ analog and control peptides.

	Structural similarity RMSD				Docking features				%PPII		# rotatable bonds		Log P		K_D_ (μM)		LD_50_ (mM)		% apoptosis		Overall rank
	WT GILZ		PPII peptide		%TA-1		%TA-2		>25	<25	<10	>10	<5	>5	<3	>3	>100	<100	<25	>25	
	<1	>1	<1	>1	>25	<25	>25	<25													
	1	0	1	0	1	0	1	0	1	0	1	0	1	0	1	0	1	0	1	0	
GA-1	1		1		1			0		0	1		1		1		1		1		8
GA-2	1		1		1			0	1		1		1			0		0	1		7
CP-1	1			0	1			0	1			0	1			0	1		1		6
CP-2		0		0	1		1		1			0		0		0		0	1		4
CP-3	0			0	1			0			1		1			0		0		0	3

## 4. Discussion

There is increasing interest in developing disease modifying agents for neurodegenerative diseases based on molecular pathogenesis. Increased NF-κΒ p65 secondary to aging and environmental stimuli contribute significantly to the inflammation and degeneration in AD [1, 2, 16]. Synthetic compounds including terpenoids like adenanthin and resveratrol or other sirtuin activators interact with NF-κΒ p65, suppress inflammation and cytotoxicity in AD models [61]. As opposed to high throughput screening, computational design of interface peptide mimics followed by functional evaluation represents an efficient method in the drug design and discovery process [28, 29]. Here we report the design and physicochemical characteristics of peptide analogs of the GILZ:p65 interface, show that select analogs bind p65-TAD and suppress Aβ induced toxicity in human fetal brain cells exhibiting potential therapeutic value for AD.

In human interactome, preponderant transient intermolecular interactions are mediated by proline rich epitope of one protein binding the aromatic residue rich flat interface of the second protein. Often the proline rich epitope adopts an extended PP_II_ helical conformation that behaves as an adaptable glove in obtaining the correct binding orientation [31, 52]. Here it is pertinent to note that the template based modeling suggested that the p65 binding domain of GILZ exhibits a PP_II_ helical conformation and that the p65-TAD is unstructured or flat. Mutational analyses identified ^120^PEA(S)P^124^ of GILZ as hot spot residues for interacting with NF-κΒ p65[24, 26]. In the GILZ:p65-TAD complex, the critical proline of GILZ, ^120^P exhibits φ and ψ angles of -67° ±5° of 142.5° ± 15° respectively and is in close proximity with the conserved phenylalanines (^534^F, ^542^F) of p65-TAD. Collectively, these observations suggest that the GILZ:p65-TAD complex represents a druggable target for development of specific therapeutic leads.

Incorporating rational substitutions in the polyproline motif of GILZ in the context of the p65-TAD interface we designed multiple peptide analogs of GILZ or GA. Molecular superposition is one of the most important means to interpret the relations between three-dimensional structures. The low RMSD upon superimposition with experimental PP_II_ and wild type GILZ suggests that the select GA represent true structural mimic of the p65 binding domain of GILZ. Significantly docking analyses showed that the top ranked GA exhibited >20% contact with the functionally critical p65-TAD residues (F^534^, F^542^) in >90% of docked solutions.

An important advantage of proline-rich motif at interface of transient intermolecular interactions is the weak binding kinetics without compromising affinity. Furthermore, it allows for introduction of small changes in the sequence of the motif or its binding domain to mediate large changes in the affinity of the interaction [31, 62, 63]. We observed that the GA-1 and GA-2 exhibited greater affinity for binding r-p65 than the full length r-GILZ. Previously, proline rich peptides that bind Src homology 3 binding domain or the transcription factor human estrogen receptor alpha or the cell surface CD80 ligand have been shown to exhibit dissociation constant (K_D_) in the micromolar range and inhibit protein:protein interactions [64, 65].

Functionally, GA-1 and GA-2 inhibited metabolic activity and suppressed cytokine responses in activated human brain cells suggesting a protective potential against Aβ induced pathology. Further we observed that GA-1 significantly suppressed caspase-3 and caspase-9 transcripts in Aβ induced human brain cells. Considerable evidence suggests that the caspases play active role in Aβ_1–42_ induced neurotoxicity. Multiple caspases including caspase-3 and caspase-9 are transcriptionally elevated in AD [66, 67]. Caspase-3 has been shown to cleave APP, thereby enhancing Aβ plaque formation and toxicity. In AD transgenic mouse model that overexpress human mutant APP, Aβ accumulation has been shown to lead to aberrant caspase-3 activation triggering a cascade of down-stream signaling events leading to synaptic loss and behavioral changes [67, 68]. Caspase-9 has been shown to induce apoptosis by directly cleaving APP or indirectly by triggering caspase-3 cleavage in AD [66, 69]. Many of the NF-κΒ antagonists including peroxisome proliferator-activated receptor-gamma agonists and anti-oxidants such as melatonin have been shown to suppress Aβ-induced caspase-3 activity [70, 71]. Collectively, the ability to suppress Aβ induced inflammatory and cytotoxic responses in human fetal brain cells support therapeutic potential for GA-1 and GA-2 in AD.

Many peptide drugs including copaxone, leuprolide acetate/goserelin (peptide antagonists of GnRH receptor), octreotide (a cyclic octapeptide mimicking natural hormone somatostatin) and glucagon like peptide-1(GLP-1) analog have been evaluated in models of AD substantiating the credibility of peptide drugs for AD [59, 60, 66]. Proline rich PP_II_ helical peptides such as apidaecin, oncocin and drosocin have shown to exhibit significant influx into CNS and distribution within the brain parenchyma [67]. Hence PGA is likely to cross the blood brain barrier and reach optimal concentration in the brain to be clinically efficient. Significantly, the estimated LD_50_ for GA-1 and GA-2 as determined by the method of Speilmann et al. is within the range of these peptide drugs [46]. Furthermore the ability of GA-1 and GA-2 to suppress Aβ induced inflammatory and cytotoxic responses in human fetal brain cells suggests potential as AD therapeutic agents.

## 5. Conclusion

**Fig 8:**
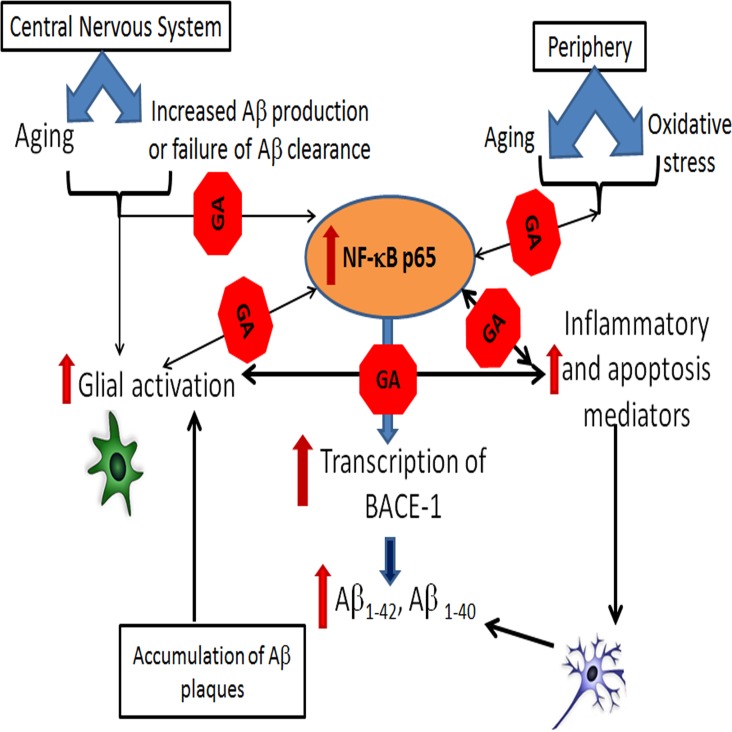
Schematic representation of pathological mechanisms of AD and points of intervention by GA: Increased oxidative stress and other age related changes upregulate NF-κΒ p65 which in turn increase transcription of beta site amyloid precursor protein cleaving enzyme-1 (BACE-1) leading to generation and accumulation of Aβ peptides in the CNS parenchyma. Glial cells exposed to Aβ peptides exhibit increased p65 activation and secrete inflammatory and apoptosis mediators. Affected neurons upregulate Aβ peptides and the vicious cycle of Aβ deposition, inflammation and neuronal apoptosis leads to AD. GILZ analogs (GA) by virtue of binding activated NF-κΒ p65 blocks Aβ generation and suppressing inflammation, thereby ameliorating AD pathology.

The goal of biological therapies is to restore healthy balance by targeting specific molecules that are critical for mediating or perpetuating imbalanced cellular responses. In recent years peptide-based drugs have gained considerable value in the discovery phase of drug development, in particular in the design of interface mimotopes [28, 29]. The functionally active peptides are amenable to further modifications into peptidomimetic compounds or small molecules with improved pharmacokinetic properties. In this context, the low molecular weight GA-1 and GA-2 can act as lead compounds in the development of specific small molecule inhibitors of NF-κΒ p65 with significant therapeutic potential for chronic neurodegenerative diseases including AD (Fig 8). Since the GA peptides exhibit similar hydrophobicity, hydropathicity, hydrophilicty and amphipathicity, the cellular uptake is likely to be equivalent. Evaluation of in-vivo stability and pharmacokinetics will be included in futures studies.

## Supporting Information

S1 Doc IRB: 1106006166- Lahiri D: Non-Human Subject Research. (PDF)

S2 Doc IBC 1623 CR15 Lahiri Final. (PDF)
