# Supplementary material for: Novel Nuclear Factor-KappaB Targeting Peptide Suppresses β-Amyloid Induced Inflammatory and Apoptotic Responses in Neuronal Cells
Source: PLoS One. 2016 Oct 20;11(10):e0160314. doi: 10.1371/journal.pone.0160314 (PMC5072831; doi:10.1371/journal.pone.0160314)
Supplement: S1 Doc — (PDF) [file pone.0160314.s001.pdf]

INDIANA UNIVERSITY INSTITUTIONAL REVIEW BOARD (IRB)

APPLICATION FOR NON-HUMAN SUBJECTS RESEARCH
(RESEARCH NOT SUBJECT TO FDA OR COMMON RULE DEFINITIONS OF HUMAN SUBJECTS RESEARCH)

IRB TRACKING NUMBER: 1106006166

Please type only in the gray boxes. To mark a box as checked, double-click the box, select "checked", and click "OK".

SECTION I: PERSONNEL INFORMATION

Principal Investigator:

Name *(Last, First, Middle Initial)*: **Lahiri, Debomoy K**
Department: Psychiatry Phone: 317-274-2706 E-Mail: dlahiri@iupui.edu
Fax: 317-274-1365 Address: 791 Union Dr; Indianapolis; IN 46202

Additional Study Contact:
Name: Balmiki Ray/ Justin M. Long Phone: 317-274-0063 E-Mail: rayb@iupui.edu ; jusmlong@iupui.edu

Student Contact, if this is a student protocol: _____ Phone: _____ Email: _____

Project Title: **Human neuron cultures for gene regulation and drug mechanism studies related to Alzheimer's disease and othet brain disorders.**

Sponsor/Funding Agency: NIH PI on Grant: Dr. D. K. Lahiri

Sponsor Protocol #/Grant #: 5R01AG018884-09 Period: from: 06/15/11 to 06/14/2012

Sponsor Type: ☒ Federal ☐ State ☐ Industry ☐ Not-for-Profit ☐ Unfunded ☐ Internally Funded

Grant Title (if different from project title): Cholinesterase Inhibitors in Alzheimer's Disease

SECTION II: PROJECT TYPE

Refer to the **IU IRB Guidance on Determining When an Activity Requires Submission to the Indiana University IRB** available on the IU Human Subjects Office website for additional information.

☐ **Project meets the definition of human subjects research; however, Indiana University is not considered engaged in this research** in accordance with the Office for Human Research Protections (OHRP) Guidance on Engagement of Institutions in Human Subjects Research available at http://www.hhs.gov/ohrp/humansubjects/guidance/engage08.html.

☐ **Research Involving Data on Decedent PHI**. Please indicate that the following criteria are satisfied:
- ☐ The use is solely for research on the identifiable health information of decedents.
- ☐ The PHI sought is necessary for the purposes of the research; and
- ☐ Upon request, the covered entity disclosing the data may require the investigator to provide documentation of the death of the individual(s) about whom information is being sought.

☐ **Limited Data Set**. The research uses or discloses PHI as a limited data set for research purposes. This project type may only be selected if the following is true: Your data set excludes 16 specified identifiers that are listed in the regulations, including: name, street address, telephone and fax numbers, e-mail address, social security number, certificate/license number, vehicle identifiers and serial numbers, URLs and IP addresses, and full face photos and other comparable images. The limited data set could include the following identifiable information: admission, discharge, and service dates, date of death, age (including age 90 and older), and five digit zip code.

Please indicate from where the data will be obtained:
- ☐ The data will be provided from a covered entity (e.g. division, department, or practice plan) separate from that of the investigator. **NOTE:** A data use agreement must be established between the entity(ies) providing the data and the investigator. See the Confidentiality and Privacy SOP for additional information.
- ☐ The data will be obtained from within the investigator's own covered entity (e.g. his/her own data or that of the department). No data use agreement is required.
- ☐ Other, please explain:

☐ **De-Identified Health Information**. The research involves the use or disclosure of de-identified health information.

This project type may only be selected if the following is true: The health information excludes all of the following: (1) Name; (2) All geographic subdivisions smaller than a state, including street address, city, county, precinct, zip codes if the geographic unit of combining all the same three initial digits contains more than 20,000 people; (3) All elements of dates (except year) for dates directly related to an individual, including birth date, admission date, discharge date, date of death; and all ages over 89 and all elements of dates (including year) indicative of such age, except that such ages and elements may be aggregated in a single category of age 90 or older; (4) Telephone numbers; (5) Fax numbers; (6) Electronic mail addresses; (7) Social security numbers; (8) Medical record numbers; (9) Health plan beneficiary numbers; (10) Account numbers; (11) Certificate/license numbers; (12) Vehicle identifiers and serial numbers, including license plate numbers; (13) Device identifiers and serial numbers; (14) Web universal resource locators (URLs); (15) Internet protocol (IP) address numbers; (16) Biometric identifiers, including finger and voice prints; (17) Full face photographic images and any comparable images; and (18) Any other unique identifying number, character, or code.

☒ **Coded Private Information or Biological Specimens**. The research involves only coded private information or specimens. To qualify for this type of review, the private information or specimens cannot be linked to specific individuals by the investigator(s) either directly or indirectly through coding systems. To qualify, both of the following conditions must be met:

☒ The private information or specimens were **not** collected specifically for this proposed research project through an interaction or intervention with living individuals. **NOTE:** If this condition is not met, then your research involves human subjects and requires a human subjects research submission.

AND

☒ The investigator(s) cannot readily ascertain the identity of the individuals to whom the private information or specimens pertain because: (mark which option(s) applies)

- ☐ The key to decipher the code will be destroyed before the research begins.
- ☐ The investigator(s) and the holder of the key will enter into an agreement prohibiting the release of the key to the investigator(s) under any circumstances, until the individuals are deceased.
- ☒ Other. Please explain: The supplier of the samples will not display the code to the PI of Indiana University School of Medicine

For additional information on research with coded private information or biological specimens, please refer to the OHRP Guidance on Research Involving Coded Private Information or Biological Specimens (October 16, 2008) at: http://www.hhs.gov/ohrp/policy/engage08.html.

SECTION III: PROJECT DESCRIPTION

1. Provide a brief description, in lay terms, of the purpose of the proposed project and the procedures to be used.

Postmortem brain samples from aborted fetus will be obtained from the Birth defect Research Laboratory at the University of Washington, Seattle. This laboratory has been funded by NIH for a prolong period of time. Brain samples are collected from the postmortem fetus (9-13 weeks old). The samples will be shipped to Indiana University School of Medicine suspended in tissue culture medium. We will prepare neuron culture from the brain samples. Birth defect Research Laboratory will not send any information about the fetus to us. Our work is based on our present studies in the same area using the primary rat neuronal culture and *in vivo* models (Bailey & Lahiri 2006, Ray *et al.* 2009). We will extend our research from rodent neuronal cultures to primary human neuronal culture and this will have immense significance. Till date, only few drugs are being used in the treatment of Alzheimer's disease (AD). However, none of these drugs can fully restrict/cure the disease process (Ray *et al.* 2011). This warrants development of newer drugs in the treatment of AD. We have recently observed that some cholinesterase inhibitors (ChEI) and some plant derived molecules have neuroprotective properties in cultured neuronal cell lines, rodent neuronal culture and *in* vivo (Bailey & Lahiri 2010, Ray *et al.* 2010). However, the effects of these compounds in primary human neurons are yet to be evaluated.
Further, small non coding RNAs (microRNAs) thought to be involved in several neurodegenerative disorders including AD. We have recently observed potential effects of one of the microRNAs; miR101 in decreasing the levels of Alzheimer's amyloid precursor protein (APP) in HeLa and glioma cells (Long & Lahiri 2011). We will also evaluate roles of several microRNAs in regulating AD pathology using the primary human culture.
We will comply with the biohazard regulations of Indiana University in preparing the neuron cultures.

2. Provide a list of all data points that will be collected below or attach a data collection sheet.

We will perform the following:

1. Prepare neuron culture.
2. Treat the neurons with different drugs such as, ChEI, NMDA receptor antagonists (memantine, Mk801), polyphenols etc. and evaluate their effects in Alzheimer's markers such as levels of APP, amyloid beta peptide, phosphorylated tau, synaptic markers.

Treatment of human neurons	Measurement	Expected outcome
ChEIs , such as rivastigmine, phenserine; NMDA antagonists, such as MK-801 and memantine.	APP, Aβ, synaptic proteins	Some ChEI and memantine will decrease the levels of APP and Aβ; they are also expected to increase synaptic markers
Curcumin, AGE, SAC, NAC etc	APP, Aβ, inflammatory markers etc	Polyphenols will decrease the levels of APP and Aβ; they are also expected to ameliorate neuroinflammation.

3. Evaluate roles of specific microRNAs in modulating important proteins related to Alzheimer's disease.

Transfection of human neurons	Measurement	Expected outcome
miR-101	APP, Aβ	Decrease the levels of intracellular APP and Aβ peptides
miR -153	APP, Aβ	Decrease the levels of intracellular APP and Aβ peptides
miR 346	APP, Aβ	Increase the levels of intracellular APP and Aβ peptides

Statement of Principal Investigator. By submitting this form, the Principal Investigator acknowledges that he/she has personally reviewed this report and agrees with the above assessment.

SECTION IV: IRB APPROVAL

☒ Accepted

☐ Denied

☐ Separate human subjects application must be submitted.

☐ Project does not meet ethical principles.

☐ Other action required:____

Authorized Signature: Kara Brocious Date: 6/21/11

Printed Name: KARA BROCIOUS

References

Bailey, J. A. and Lahiri, D. K. (2006) Neuronal differentiation is accompanied by increased levels of SNAP-25 protein in fetal rat primary cortical neurons: implications in neuronal plasticity and Alzheimer's disease. *Ann N Y Acad Sci,* **1086,** 54-65.

Bailey, J. A. and Lahiri, D. K. (2010) A novel effect of rivastigmine on pre-synaptic proteins and neuronal viability in a neurodegeneration model of fetal rat primary cortical cultures and its implication in Alzheimer's disease. *J Neurochem,* **112,** 843-853.

Long, J. M. and Lahiri, D. K. (2011) MicroRNA-101 downregulates Alzheimer's amyloid-beta precursor protein levels in human cell cultures and is differentially expressed. *Biochem Biophys Res Commun,* **404,** 889-895.

Ray, B., Bailey, J. A., Sarkar, S. and Lahiri, D. K. (2009) Molecular and immunocytochemical characterization of primary neuronal cultures from adult rat brain: Differential expression of neuronal and glial protein markers. *J Neurosci Methods,* **184,** 294-302.

Ray, B., Bisht, S., Maitra, A. and Lahiri, D. K. (2011) Neuroprotective and Neurorescue Effects of a Novel Polymeric Nanoparticle Formulation of Curcumin (NanoCurc) in the Neuronal Cell Culture and Animal Model: Implications for Alzheimer's disease. *J Alzheimers Dis,* **23,** 61-77.

Ray, B., Chauhan, N. B. and Lahiri, D. K. (2010) Oxidative insults to neurons and synapse are prevented by Aged Garlic Extract (AGE) and S-allyl-L-Cysteine (SAC) treatment in the neuronal culture and APP-Tg mouse model. *J Neurochem.*
