# Supplementary material for: Novel Nuclear Factor-KappaB Targeting Peptide Suppresses β-Amyloid Induced Inflammatory and Apoptotic Responses in Neuronal Cells
Source: PLoS One. 2016 Oct 20;11(10):e0160314. doi: 10.1371/journal.pone.0160314 (PMC5072831; doi:10.1371/journal.pone.0160314)
Supplement: S2 Doc — (PDF) [file pone.0160314.s002.pdf]

Institutional Biosafety Committee (IBC)
Annual Continuing Review

PROTOCOL #	1623 CR15
CR APPROVED:	11/30/2015
CR EXPIRES:	11/30/2016
OFFICE USE ONLY	

Section I. Investigator Information

Principal Investigator:	**Debomoy K. Lahiri**		
Department:	**Psychiatry**		
Email:	**dlahiri@iupui.edu**		
IBC Protocol #:	**1623**		
Originally Approved On:	11/8/2013		
Approved Biosafety Level:	**2 w/ BL3 practices**		
Project Title:	**Regulation of Gene Expression in Alzheimer's Disease**		
Funding Agency(s):	**NIH**	☐ VA Affiliated:	
Office Location:	**NB200C**	Office Phone #:	**274-2706**
All Rooms Used for Protocol:	**NB201** B	Lab Phone #:	**274-0063**

IMPORTANT: THIS FORM MUST BE TYPED AND SUBMITTED AS A WORD DOCUMENT OR NEATLY PRINTED AND RECEIVED BY RESEARCH COMPLIANCE ADMINISTRATION THREE WEEKS PRIOR TO THE EXPIRATION DATE. EVEN IF YOU CHOOSE TO TERMINATE THIS STUDY, IT IS IMPERATIVE THAT YOU COMPLETE AND RETURN THIS FORM.

Section II. Current Study Status

☒ Ongoing

Date study was initiated:	**12/17/2014**
Projected date of completion:	**12/16/2019**

☐ Project not yet initiated

Projected date of initiation:	

☐ Project was discontinued/completed*

Date study was discontinued:	

* If checked, this project will be removed from IBC active files.

Section II. Current Study Status

1. Please review the list of personnel included in the continuing review reminder email. Have there been changes in personnel in the past 12 months that were not part of the original proposal?

☒ Yes. Has an amendment describing these changes been approved by the IBC?

☐ Yes. Minor amendment number(s):

X No. Submit an **IBC Amendment** form with this continuing review.

☐ No

**Note: All personnel must complete required training before beginning work on an IBC protocol. Please visit the IBC Training page for more information.*

2. Please review the list of locations (i.e., lab and/or storage space) included in the continuing review reminder email. Has the location in which project work is performed been changed in the past 12 months?

- ☐ Yes. Has an amendment describing these changes been approved by the IBC?
  - ☐ Yes. Minor amendment number(s):
  - ☐ No. Submit an **[IBC Amendment]** form with this continuing review.
- ☒ No

3. Have any new organisms been added to this protocol in the past 12 months?

- ☐ Yes. Has an amendment describing these changes been approved by the IBC?
  - ☐ Yes. Major amendment number(s):
  - ☐ No. Submit an **[IBC Amendment]** form with this continuing review.
- ☒ No

4. Have any new strains of previously approved organisms been added to this protocol in the past 12 months?

- ☐ Yes. Has an amendment describing these changes been approved by the IBC?
  - ☐ Yes. Minor amendment number(s):
  - ☐ No. Submit an **[IBC Amendment]** form with this continuing review.
- ☒ No

5. Have there been any major changes (i.e., major amendments) to this protocol within the past 12 months?

- ☐ Yes. Has an amendment describing these changes been approved by the IBC?
  - ☐ Yes. Major amendment number(s):
  - ☐ No. Submit an **[IBC Amendment]** form with this continuing review.
- ☒ No

6. At any time during the course of the research, has the Biosafety Level changed?

- ☐ Yes. Original Biosafety Level: ___________
  Current Biosafety Level: ___________
- ☒ No

7. Have there been significant problems, violations of the *NIH Guidelines*, or serious accidents within the past year, or if this is a human gene therapy study, have there been any serious adverse events within the past year?

- ☐ Yes. Have these problems/violations/accidents/events been reported to the IBC?
  - ☐ Yes. Date of submission to IBC:
  - ☐ No. Provide a thorough description:
- ☒ No

Return this continuing review and any associated documentation to the Office of Research Compliance:

IUPUI: ibcbhc@iupui.edu
IUB & Regionals: iubibc@indiana.edu

For questions, contact Research Compliance Staff at 812-856-0656. Retain a copy of this form for your records.

This project is being conducted as originally submitted, or all amendments to this proposal have been previously approved, or are attached. I agree to abide by the federal guidelines in conducting all work using recombinant DNA molecules.

___ Debomoy K. Lahiri ____ __11/30/15_______________

Principal Investigator Signature

Date

☐ Type name above and check for electronic signature

IBC OFFICE USE ONLY

Emily Winters

Digitally signed by Emily Winters
DN: cn=Emily Winters, o=Indiana University, ou=Office of Research Compliance, email=ejwinter@iu.edu, c=US
Date: 2015.11.30 12:45:04 -05'00'

Office of Research Compliance

Continuing Review Final Approval Date
